# Incidence and number of fragility fractures of the hip in South Africa: estimated projections from 2020 to 2050

**DOI:** 10.1007/s00198-022-06525-5

**Published:** 2022-08-13

**Authors:** Samuel Hawley, Sapna Dela, Anya Burton, Farhanah Paruk, Bilkish Cassim, Celia L Gregson

**Affiliations:** 1 Musculoskeletal Research Unit, Translational Health Sciences, Bristol Medical School, University of Bristol, Bristol, UK; 2 Department of Internal Medicine, Edendale Hospital, Pietermaritzburg, South Africa; 3 Department of Rheumatology, School of Clinical Medicine, University of KwaZulu-Natal, Durban, South Africa; 4 Department of Geriatrics, School of Clinical Medicine, University of KwaZulu-Natal, Durban, South Africa; 5 SAMRC/Wits Developmental Pathways for Health Research Unit, Department of Paediatrics, School of Clinical Medicine, University of the Witwatersrand, Johannesburg, South Africa

**Keywords:** Hip fracture, Incidence, South Africa, Sub-Saharan Africa, Osteoporosis, Epidemiology

## Abstract

**Purpose:**

A better understanding of the burden of fragility fractures in sub-Saharan Africa is needed to inform healthcare planning. We aimed to use recent hip fracture incidence data from South Africa (SA) to estimate the future burden of hip fracture for the country over the next three decades.

**Methods:**

Hip fracture incidence data within the Gauteng, KwaZulu-Natal and Western Cape provinces of SA were obtained from patients aged ≥40 years with a radiograph-confirmed hip fracture in one of 94 included hospitals. Age-, sex- and ethnicity-specific incidence rates were generated using the 2011 SA census population for the study areas. Incidence rates were standardised to United Nations (UN) population projections, for the years 2020, 2030, 2040 and 2050, and absolute numbers of hip fractures derived.

**Results:**

The 2767 hip fracture patients studied had mean (SD) age 73.7 (12.7) years; 69% were female. Estimated age- and ethnicity-standardised incidence rates (per 100,000 person-years) for the overall SA population in 2020, were 81.2 for females and 43.1 for males. Overall projected incidence rates were discernibly higher by the year 2040 and increased further by the year 2050 (109.0 and 54.1 for females and males, respectively). Estimates of the overall annual number of hip fractures for SA increased from approximately 11,000 in 2020 to approximately 26,400 by 2050.

**Conclusion:**

The hip fracture burden for SA is expected to more than double over the next 30 years. Significant investment in fracture prevention services and inpatient fracture care is likely to be needed to meet this demand.

## Introduction

Population ageing is a global reality, with sub-Saharan Africa predicted to experience the most rapid population growth this century, reaching an expected 157 million ≥60 year olds by 2050 (1). Ageing is associated with an increased burden of comorbidities, including a high burden of musculoskeletal conditions (2). This rapid growth in non-communicable diseases poses a particular challenge in sub-Saharan Africa where health systems have traditionally been focussed on tackling infectious diseases and maternal and childhood mortality (3), rather than age-related comorbidity.

In the context of the epidemiological transition underway in sub-Saharan Africa, data on the prevalence of osteoporosis are now emerging (4), indicating osteoporosis is more common than has hitherto been appreciated (5). Hip fracture has been described as the international barometer of the impact of osteoporosis (6). Hip fractures in high-income settings have considerable impact on morbidity, mortality and healthcare costs (7), with recent data from South Africa (SA) suggesting similar healthcare costs (8), long hospital stays and high mortality (9). Although incidence of fragility fracture in the region has historically been considered to be low, recent data suggest such fractures are actually more common than previously reported (10, 11).

Despite the need to invest in prevention and treatment of non-communicable diseases across sub-Saharan Africa (3), key epidemiological data to inform policy and planning of fragility fracture management in the region are currently lacking (12). The aim of the present study was to utilise recently collected hip fracture incidence data for SA and combine these with future population projections for the country to improve understanding of the predicted hip fracture burden over coming decades.

## Methods

### Study population and data collection

Data were obtained from a previously published South African hip fracture incidence study conducted between 1^st^ April 2017 and 31^st^ March 2018 (10). In brief, data sampling took place in three SA provinces: Gauteng (GP), Western Cape (WC) and KwaZulu-Natal (KZN). These provinces collectively make up over 55% of the SA population and provided adequate representation to study the four population ethnicity groups as characterised in the 2011 SA census: Black African, Coloured (a term used in SA to describe a combination of two or other ethnic groups (13)), Indian and White. Age, sex and ethnicity distributions were readily available for these provinces (14) and well-developed and accessible health care infrastructure enabled reliable hip fracture identification.

Across the three provinces, a total of eight districts were selected in which all eligible patients with a fragility fracture of the hip were prospectively identified. These districts were: eThekwini and Msunduzi municipalities (KZN); Johannesburg Metropolitan and Sedibeng municipalities (GP) and Cape Metropole, West Coast, Cape Winelands and Overberg (WC). Within the 94 identified hospitals (25 [26.6%] public sector and 69 [73.4%] private) in the included districts, all hospital managers, administrators, affiliated orthopaedic surgeons and nursing staff were sensitised to the study and requested to prospectively identify all hip fracture admissions. Cases were also identified through admission registers in the wards. Study fieldworkers identified key personnel with whom regular contact was made. All incident hip fracture patients aged ≥40 years who presented within the study period with a fragility fracture (from standing height or less) of the hip, neck of femur or trochanter were enrolled in the study, provided their pre-admission residence was within one of the eight study districts; that the hip fracture was their first such occurrence in the study period and not a readmission; and that the fracture was radiographically confirmed. High velocity trauma, pathological and peri-prosthetic fractures were excluded.

### Statistical analysis

Demographic, hospital and seasonality data were summarised using descriptive statistics. The sex-, ethnicity- and age-specific incidence of hip fracture was calculated for the study sample using 5-year age categories (one category was used for those aged 85+ years as per the SA census). Incidence was calculated by dividing the fracture count in each stratum by the population at risk, as reported in the 2011 SA census. In this way the total 2011 census population aged ≥40 years within the eight study districts (4,034,153 people; 29.5% of the total SA population aged ≥40 years) was used as the denominator for calculating the total hip fracture incidence. Incidence was expressed as per 100,000 person-years (PYs).

Age- and sex-stratified population projections for SA were obtained from the United Nations (UN) Department of Economic and Social Affairs population prospects 2019 medium fertility estimates for the years 2020, 2030, 2040 and 2050 (15). These were further stratified by ethnicity assuming distributions in the SA 2011 census remained stable (14, 16). In initial explorative analysis of the reliability of the UN future projections, the age- and sex-stratified UN estimated population for the year 2011 was compared to the 2011 SA census population. Given a deficit was found in the UN estimated population aged ≥80 years, a correction factor was derived to account for this deficit in the future population projections (Supplementary Figure 1).

Future hip fracture incidence rates were generated by applying the observed incidence rates from the study sample to the age distribution of the (corrected) UN population projections at each future timepoint. The overall sex-stratified rates were standardised to the ethnicity- and age-structure of UN population projections at each timepoint, whilst corresponding rates further stratified by ethnicity were standardised for age only. These future incidence rate estimates were then used to derive the actual number of hip fractures estimated at each timepoint: overall, stratified by ethnicity, and further stratified by sex. Statistical analyses were carried out using Stata v15.1.

### Sensitivity analysis

Analyses were repeated using population projections as estimated by The World Bank (17) rather than United Nations. Standardisation according to the age structure at each timepoint was performed using 5-year age categories as similar to the main analysis, except those aged 80+ years were merged into one category as this was the best granularity available from the World Bank Databank. Given a deficit was again observed in the World Bank SA population projections for those aged 80+ years, an updated correction factor was derived and applied (Supplementary Figure 2).

### Ethics and approvals

Ethical approval was obtained from the Biomedical Ethics Research Committees of the Universities of KwaZulu-Natal, Cape Town, Stellenbosch and Witwatersrand and all study sites, including approval from the Regional, Provincial and National Departments of Health as well as private health care providers. The study was conducted according to the ethical guidelines and principles of the International Declaration of Helsinki and South African Guidelines for Good Clinical Practice. In addition to the patients who were able to consent, ethical approval was obtained to record demographic data for individuals who did not or were unable to consent.

## Results

A total of 2,767 eligible hip fractures were included in incident rate analysis. Table 1 contains summary patient characteristics: 69.2% were women and mean age was 73.7 years (S.D. 12.7 [range: 42 to 104]). The most common ethnicity was White (40.9%), followed by Black African (26.4%), Coloured (18.7%) and Indian (14.0%). More fractures occurred in winter (28.8%) than in summer months (21.6%), while the majority of patients were identified within the public sector (66.3%). Similar numbers of fractures were identified in KZN (39.1%) and WC (40.9%), while the number in GP was approximately half of those (20.0%).

**Figure 1:**
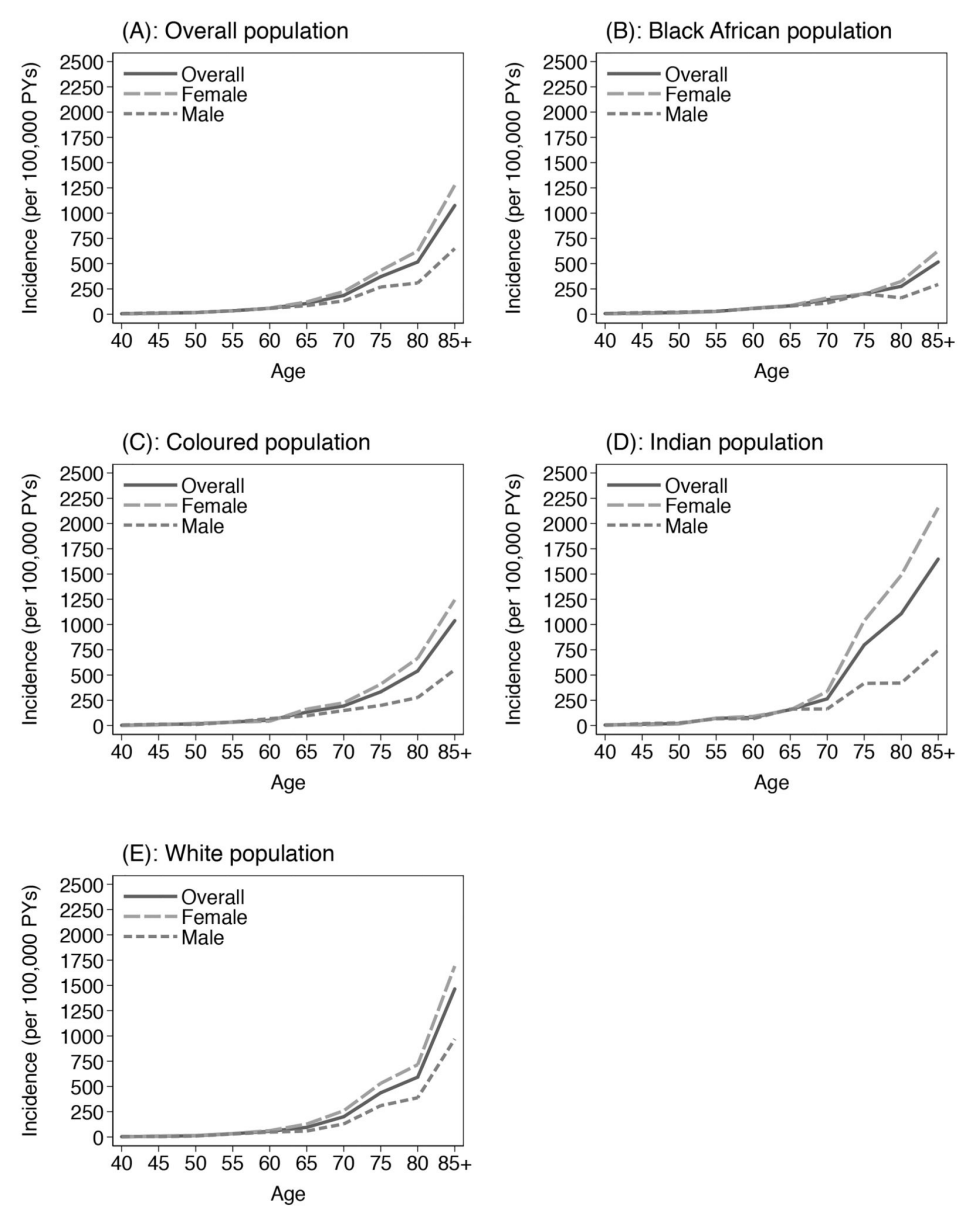
Contemporary age-specific hip fracture incidence rates in study areas: overall and stratified by sex and ethnicity

The total hip fracture incidence (per 100,000 PYs) in the study areas was 68.6; 87.5 among women and 46.2 among men (Supplementary Table 1). Total incidence rates by ethnicity were: 129.8 (White), 37.9 (Black Africans), 58.3 (Coloured) and 111.7 (Indian) (Supplementary Table 1). Age-specific hip fracture incidence rates in the study areas for the overall population and stratified by sex and ethnicity are presented in Figure 1. As expected, these show monotonic increases in fracture rates with age and higher rates among women, which were particularly prominent among the Indian population but less prominent among the Black African population.

**Figure 2:**
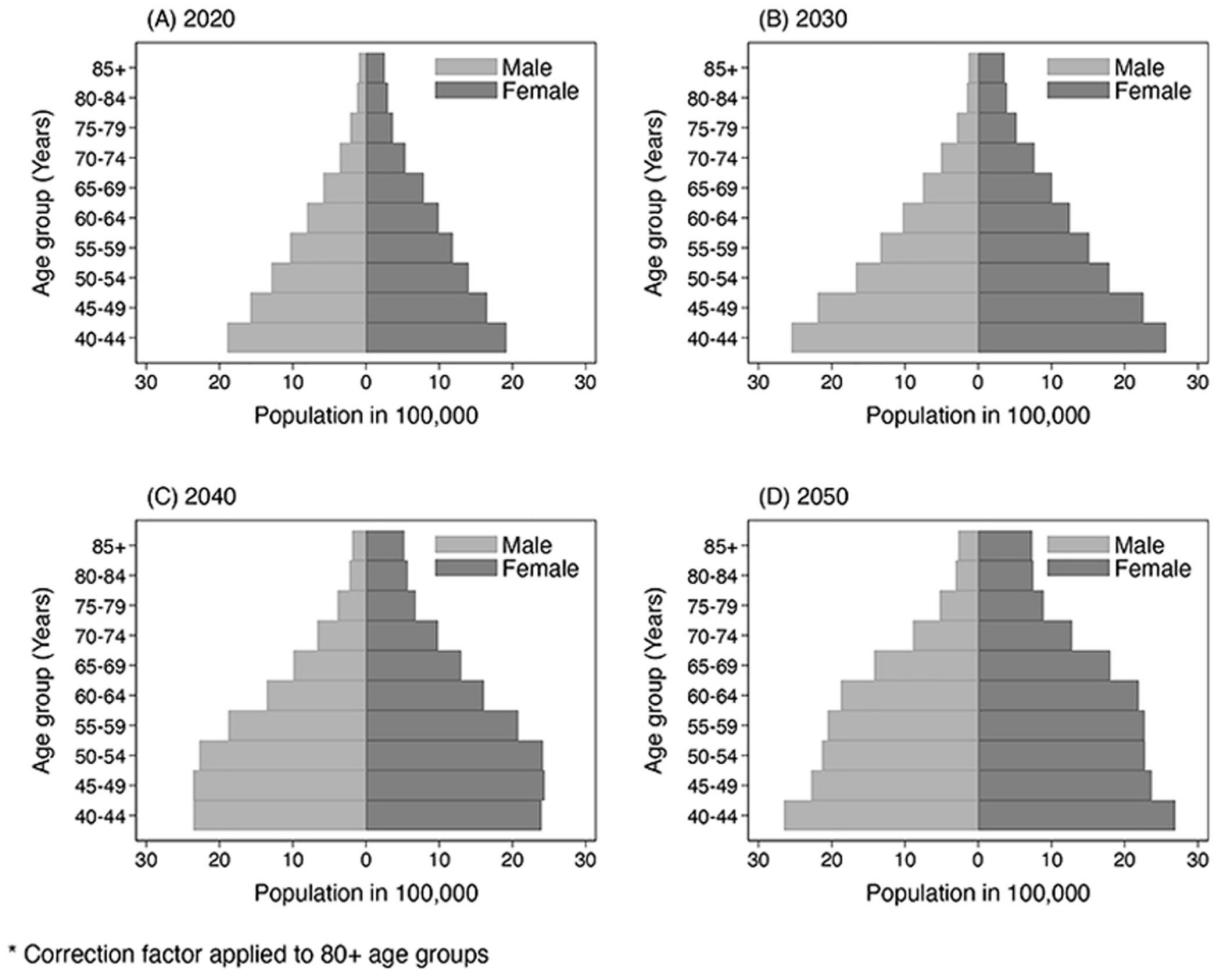
United Nations estimated population prospects for South Africa (2020 to 2050)*: stratified by age and sex

The UN population projections for SA among those aged ≥40 years are shown in Figure 2, which demonstrate a notable ageing of the population over time as life expectancy improves. For example, while little discernible growth is expected between 2030 and 2050 among 40-49 year olds, there is an approximate doubling in the number of 65-69 year olds (Figure 2).

**Figure 3:**
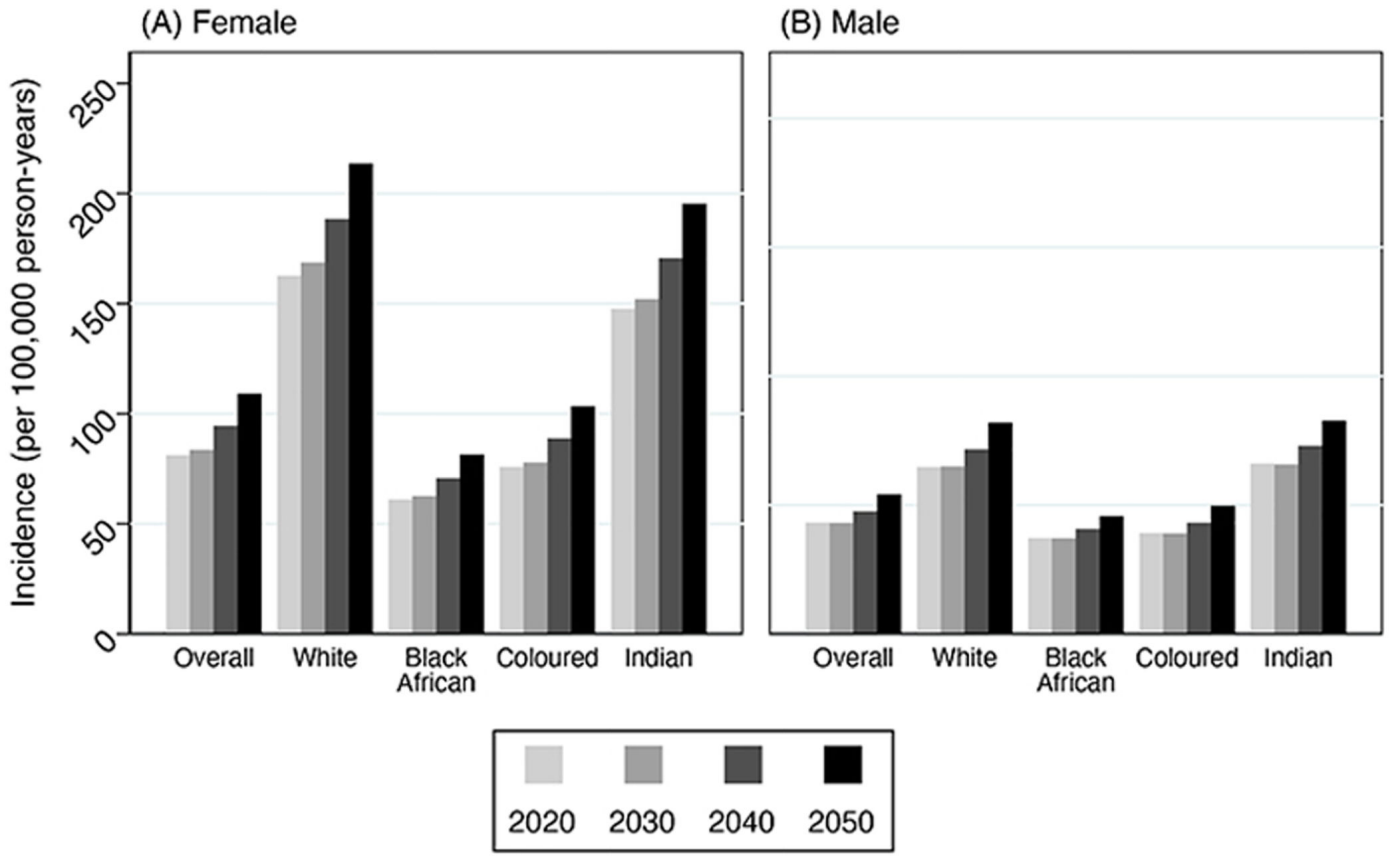
Sex-specific hip fracture incidence standardised to future South African population prospects (2020 to 2050): overall and stratified by ethnicity

The expected future incidence rates for the whole of SA are shown in Figure 3 and Supplementary Table 2. These indicate an overall trend of increasing hip fracture incidence (per 100,000 PYs) from 2020 to 2050: 81.2 rising to 109.0 amongst women, and 43.1 increasing to 54.1 amongst men. Increases are most notable from 2040 onwards. Indeed, amongst men hip fracture incidence is stable until 2040.

**Figure 4:**
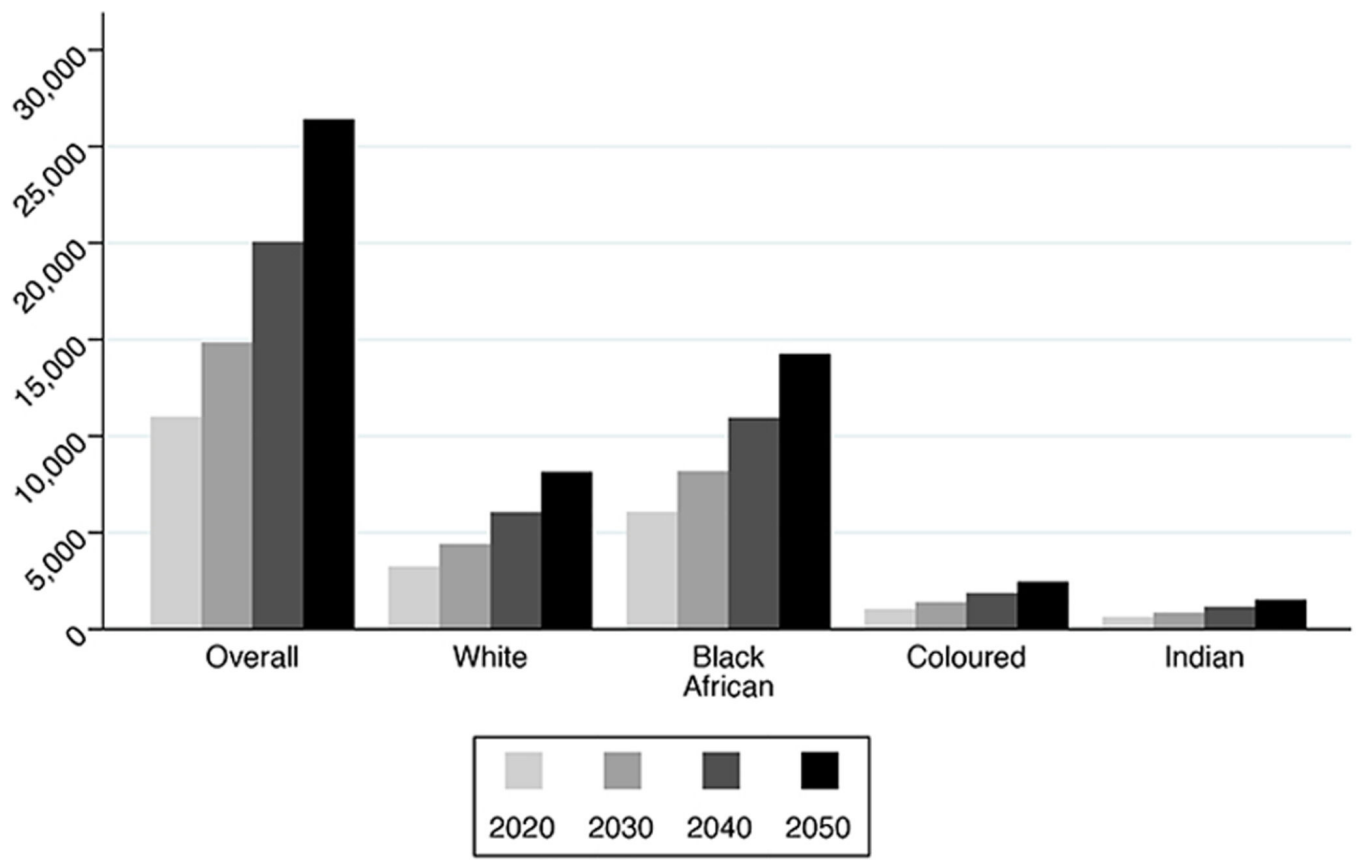
Estimated total number of hip fractures expected in South Africa (2020 to 2050): overall and stratified by ethnicity

The corresponding overall absolute number of hip fractures for the whole of SA is expected to increase from 11,005 (2020) to 14,856 (2030), 20,061 (2040) and 26,397 (2050) (Figure 4). By 2050 the absolute number of hip fractures is expected to have more than doubled among both women and men to 18,644 and 7,754, respectively (Supplementary Table 3). At each time point, the greatest number of hip fractures are predicted to occur in the Black African population (Figure 4).

Future incidence rate estimates and absolute numbers of hip fractures were very similar in sensitivity analyses using population prospects as estimated by The World Bank (Supplementary Figure 3 & Supplementary Figure 4).

## Discussion

Emerging data indicate hip fracture incidence in SA is higher than suggested by historical estimates (10, 18, 19). While still having one of the lower rates globally (20), recent findings challenge the notion of very low incidence of hip fracture for Southern Africa (12). The present study combines a large and contemporary hip fracture cohort with population projections to demonstrate that incidence in SA is expected to increase further in coming decades, with more than twice as many hip fractures expected to occur in 2050 than in 2020. By way of comparison, the expected number in 2050 will be approximately one third of those currently seen per annum in the United Kingdom (21).

Regarding our contemporary hip fracture incidence rates (as reported previously (10)), incidence was higher among women than men, and particularly so among Indian and White sub-populations which had the highest overall rates. Incidence was lowest among Black Africans, with crude rates broadly similar to those recently reported from neighbouring Botswana (22). It is worth noting we found higher rates in Black African women (43.6/100,000 PYs) than Black African men (31.1/100,000 PYs), whilst little divergence by sex was found in the Botswana study (33.2 and 33.4/100,000 for women and men, respectively), which was nearly entirely Black African (22). However, it has been noted that lifetime probability of hip fracture is over four times higher among Black Africans in SA than in Botswana, explained by higher age-specific incidence and longer life expectancy (22). White South African women have previously been reported to have approximately five times higher lifetime probability of hip fracture compared to Black South African women (23), demonstrating the dramatic in-country ethnic disparity within SA. Potential reasons for such disparity have been discussed previously (10). Lower rates among in-country Black sub-populations as compared to White sub-populations have also been reported in the UK (24) and the US (25). Likewise ethnic disparities in BMD (13) and bone strength (26) have also been reported in the US, although comparisons with Sub-Saharan African data indicate both similarities and differences, highlighting the importance of local data (13, 27).

Although we found projected incidence rates to be lowest among Black Africans, it is important to note that the expected absolute number of hip fractures is highest in this sub-population, given that the majority of the SA population is Black African (14). A large increase in hip fractures in SA, particularly among Black Africans, is likely to further exacerbate the existing need to improve clinical outcomes for these patients (9). Contemporary SA post hip-fracture mortality at 1-year (33.5%) (9) is higher than many countries (28). Furthermore, Black Africans have been shown to have the worst survival rates in SA, with 1-year post-hip fracture mortality of 41% (9) - findings which are consistent with emerging data on disparities in hip fracture care and outcomes for non-white hip fracture patients in the US (29, 30). Recent data from five SA public hospitals indicate that time from hospital admission to hip fracture surgery predicts mortality at 1-year: hazard ratio 1.02 (*p*=0.022) for each day of surgical delay (9). While previous reports have been varied in terms of achievement of prompt surgery (9, 31), it is common for hip fracture care to lack the recommended multi-disciplinary approach, with few patients investigated or treated for osteoporosis (32). Such data indicate various existing opportunities for healthcare quality improvement. Similar to many other non-communicable diseases (3), healthcare investment will be needed to expand existing service provision in sub-Saharan Africa to meet the large increase in demand for hip fracture care over coming decades.

The need for more spending on hip fracture services in SA is likely to be particularly necessary in public hospitals, demonstrated by the fact that in the present study 66% of hip fracture patients were seen in the public sector. Nationally fewer than 20% of the population are covered by a medical aid scheme (33), with coverage likely to be half of this among Black Africans (34). Data on the management and costs of hip fracture in SA are only now emerging, but our recent analysis across five public hospitals in KZN estimated mean costs of acute management following an index hip fracture to be RAND 108,525 (approximately €6,446 at an exchange rate of 0.0594) (8). By way of comparison, in the European Union (EU) each individual hip fracture is estimated to cost €13,816 on average (21).

An encouraging recent development is the new calibration of the FRAX tool using these incidence data, to create a freely available SA-specific tool with enhanced accuracy of sex- and ethnicity-specific fracture probabilities (23). This also allows risk stratification according to clinical risk factors, prior fragility fracture with or without a femoral neck *T* score. In this context, it is worth noting that while we have here estimated projections for hip fracture, more data are required to robustly model the burden of non-hip fragility fractures in SA. Further work is needed to confirm whether the ratio of hip to non-hip fracture in SA is the same as for the EU, which is approximately 1:5 (7). Although hip fractures are likely to account for the majority of the total cost of osteoporosis care, in the EU a substantial proportion (46%) of this total cost remains attributable to non-hip fractures (21). Furthermore, for the EU, it has been estimated that hip fractures alone account for approximately half the quality adjusted life years (QALYs) lost due to all fractures, indicating the substantial burden on patient’s lives caused by other fracture types. The impact of fractures on quality of life in Africa is the subject of ongoing research, with the Fractures-E3 program currently collecting data on fracture prevalence in The Gambia, Zimbabwe and South Africa (35). The Organisation of Economic Co-operation and Development (OECD) DAC list of official development assistance (i.e. aid) (36) currently includes The Gambia in the group of least developed countries globally, and Zimbabwe as another low income country based on gross national income. South Africa being a middle income country has transitioned further in terms of development (36), but has the lowest income equality in the world (37), meaning that a large proportion of the country still lives in poverty. As such, these countries being investigated in the Fractures-E3 study reflect the wide range of development within Africa.

Various limitations to the present study need to be acknowledged. Notably, denominators for the study areas were comprised of 2011 census data, which were then used to generate incidence rates. While these were the most recent available, they were nonetheless collected 6-7 years before the incidence study and were not strictly contemporaneous. This may have particular implications for the age group over 80 years, as there seems to be a greater uncertainty around population growth at the extremes of age (Supplementary Figure 1). Although a 2022 SA census is currently underway, it is unclear whether the population estimates once published will provide a more suitable denominator to that already used, given they will also not be contemporaneous. Our projections assume age-, sex- and ethnicity-specific fracture rates will be stable into the future, as has generally been observed in secular trends of hip fracture incidence in high-income countries (38). Were age-specific rates to rise over future years then our projection estimates would prove conservative. It should be mentioned that the study areas were all urban and it is possible incidence rates are different in rural areas, *e.g*., potentially being lower due to different environmental or lifestyle factors (39). This will be explored in the Fractures-E3 study, which is currently collecting data on hip fractures in rural and urban areas of The Gambia and Zimbabwe (35) and will provide insight into the incidence and risk factors for hip fracture in such low income settings. Furthermore, our analysis was not able to account for other changing trends nor identify risk factors for incidence, *e.g*., people living longer with HIV, with long-term HIV infection and treatment having deleterious effects on bone (5). We were also not able to investigate the possible role of rising rates of obesity that may offer some protection against hip fracture (although not for other fracture types) (40).

Our study has several strengths. The eight districts forming the geographical area of the study make up a large proportion of the country – nearly 30% of the whole SA population aged ≥40 years. This yielded a broadly representative sample allowing for standardisation and stratification by ethnicity which is a key factor in such a multi-ethnic country, particularly given the difference in rates of hip fracture by ethnicity. Hip fractures were radiographically confirmed by the study team who worked prospectively with all the 94 hospitals in which hip fractures were identified. Care was taken to include all public and private health facilities in the study sites to minimise socio-economic selection bias, an important study design feature. Two different sources of data on population prospects were also used for sake of comparison and ensure consistency of estimates.

To conclude, we have demonstrated the hip fracture burden for SA, whose last census population was 52 million, is estimated to more than double over the next 30 years. Incidence rates for the country are higher than traditionally thought and are expected to rise further. Significant investment in fracture prevention services and inpatient fracture care is likely to be needed to meet this growing demand.

## Supplementary Material

Supplementary material

## Figures and Tables

**Table 1:** Characteristics of hip fracture patients (N=2,767)

	Number	%
Sex		
Female	1914	69.2
Male	853	30.8
Age: mean (S.D.)	73.7 (12.7)	
Ethnic Group		
African	730	26.4
Coloured	518	18.7
Indian	387	14.0
White	1132	40.9
Province		
Gauteng	554	20.0
KZN	1081	39.1
Western Cape	1132	40.9
Public or Private Hospital*		
Public	1835	66.3
Private	931	33.7
Season of fracture		
Spring	720	26.0
Summer	598	21.6
Autumn	651	23.5
Winter	798	28.8

* Unknown for 1 patient

## Mini Abstract

Sub-Saharan Africa is undergoing rapid population ageing and better understanding of the burden of musculoskeletal conditions is needed. We have estimated a large increase in the burden of hip fractures for South Africa over the coming decades. These findings should support preparation of hip fracture services to meet this demand.
