# Supplementary material for: Incidence and number of fragility fractures of the hip in South Africa: estimated projections from 2020 to 2050
Source: Osteoporos Int. Author manuscript; Available in PMC 2026 Aug 5. (PMC7619312; doi:10.1007/s00198-022-06525-5)
Supplement: Supplementary file 1 [file EMS213984-supplement-Supplementary_material.pdf]

Supplementary Table 1: Age-specific and total incidence of hip fractures in study sample: overall and by sex and ethnic group									
Age category (years)	Female			Male			Both Female and Male		
	Number of fractures	Number at risk*	Incidence rate (per 100,000 Pys)	Number of fractures	Number at risk*	Incidence rate (per 100,000 Pys)	Number of fractures	Number at risk*	Incidence rate (per 100,000 Pys)
Overall									
40-44	17	470668	3.6	32	459139	7.0	49	929807	5.3
45-59	29	424033	6.8	54	371705	14.5	83	795738	10.4
50-54	59	356371	16.6	53	308150	17.2	112	664521	16.9
55-59	98	286816	34.2	84	241560	34.8	182	528376	34.4
60-64	134	221459	60.5	105	181982	57.7	239	403441	59.2
65-69	180	148003	121.6	99	116016	85.3	279	264019	105.7
70-74	255	113915	223.9	103	78790	130.7	358	192705	185.8
75-79	331	76546	432.4	123	45736	268.9	454	122282	371.3
80-84	311	49740	625.3	80	25883	309.1	391	75623	517.0
85+	500	39163	1276.7	120	18478	649.4	620	57641	1075.6
Total	1914	2186714	87.5	853	1847439	46.2	2767	4034153	68.6
White									
40-44	0	69371	0	4	65318	6.1	4	134689	3.0
45-59	7	65207	10.7	2	59018	3.4	9	124225	7.2
50-54	10	64798	15.4	6	59261	10.1	16	124059	12.9
55-59	21	60192	34.9	16	54057	29.6	37	114249	32.4
60-64	36	57119	63.0	25	51573	48.5	61	108692	56.1
65-69	61	47643	128.0	25	42320	59.1	86	89963	95.6
70-74	99	38055	260.1	41	31723	129.2	140	69778	200.6
75-79	147	27673	531.2	63	20274	310.7	210	47947	438.0
80-84	149	20801	716.3	49	12607	388.7	198	33408	592.7
85+	294	17375	1692.1	77	7942	969.5	371	25317	1465.4
Total	824	468234	176.0	308	404093	76.2	1132	872327	129.8
Black African									
40-44	14	258565	5.4	21	266057	7.9	35	524622	6.7
45-59	13	223477	5.8	34	196582	17.3	47	420059	11.2
50-54	24	175333	13.7	32	149935	21.3	56	325268	17.2
55-59	35	134817	26.0	34	112414	30.2	69	247231	27.9
60-64	56	92423	60.6	41	73363	55.9	97	165786	58.5
65-69	47	55371	84.9	30	37203	80.6	77	92574	83.2
70-74	68	42230	161.0	26	23774	109.4	94	66004	142.4
75-79	55	27287	201.6	26	12898	201.6	81	40185	201.6
80-84	56	17262	324.4	12	7414	161.9	68	24676	275.6
85+	86	13711	627.2	20	6803	294.0	106	20514	516.7
Total	454	1040476	43.6	276	886443	31.1	730	1926919	37.9
Coloured									
40-44	0	107522	0.0	6	93895	6.4	6	201417	3.0
45-59	7	101936	6.9	12	86502	13.9	19	188438	10.1
50-54	20	86211	23.2	8	72584	11.0	28	158795	17.6
55-59	22	65002	33.8	19	52941	35.9	41	117943	34.8
60-64	21	49013	42.8	26	38028	68.4	47	87041	54.0
65-69	47	28774	163.3	23	23646	97.3	70	52420	133.5
70-74	51	22656	225.1	23	15402	149.3	74	38058	194.4
75-79	62	15123	410.0	17	8501	200.0	79	23624	334.4
80-84	55	8251	666.6	11	3961	277.7	66	12212	540.5
85+	74	5944	1245.0	14	2529	553.6	88	8473	1038.6
Total	359	490432	73.2	159	397989	40.0	518	888421	58.3
Indian									
40-44	3	35210	8.5	1	33869	3.0	4	69079	5.8
45-59	2	33413	6.0	6	29603	20.3	8	63016	12.7
50-54	5	30029	16.7	7	26370	26.5	12	56399	21.3
55-59	20	26805	74.6	15	22148	67.7	35	48953	71.5
60-64	21	22904	91.7	13	19018	68.4	34	41922	81.1
65-69	25	16215	154.2	21	12847	163.5	46	29062	158.3
70-74	37	10974	337.2	13	7891	164.7	50	18865	265.0
75-79	67	6463	1036.7	17	4063	418.4	84	10526	798.0
80-84	51	3426	1488.6	8	1901	420.8	59	5327	1107.6
85+	46	2133	2156.6	9	1204	747.5	55	3337	1648.2
Total	277	187572	147.7	110	158914	69.2	387	346486	111.7

* Number at risk obtained from South Africa 2011 census data

Supplementary Table 2: Projected hip fracture incidence per 100,000*: by sex and ethnic group										
Year	White		African		Coloured		Indian		Overall	
	Female	Male	Female	Male	Female	Male	Female	Male	Female	Male
MAIN ANALYSIS using (corrected) United Nations estimates										
2020	162.6	64.7	61.1	37.1	75.9	39.1	147.6	66.0	81.2	43.1
2030	168.5	64.9	62.6	37.0	77.8	39.0	152.0	65.7	83.5	43.1
2040	188.4	71.6	70.7	40.7	88.8	43.1	170.6	72.9	94.5	47.6
2050	213.6	81.9	81.5	45.7	103.3	49.7	195.4	82.6	109.0	54.1
SENSITIVITY ANALYSIS using (corrected) World Bank estimates										
2020	172.6	71.4	63.1	37.8	78.7	40.1	158.6	68.0	84.9	45.0
2030	176.2	70.9	64.0	37.6	80.1	39.8	162.3	67.5	86.3	44.7
2040	197.6	78.6	72.4	41.4	91.5	44.1	183.1	75.1	97.9	49.5
2050	221.4	89.8	82.8	46.5	105.8	50.9	209.0	85.1	112.0	56.4

*Rates atandardised to age structure at each timepoint. Overall incidence rate standardised for age and ethnicity

Supplementary Table 3: Estimated number of hip fractures in South Africa (2020 - 2050)*: by ethnic group and sex					
Ethnicity	Sex	2020	2030	2040	2050
MAIN ANALYSIS using (corrected) United Nations estimates					
Overall	Female & Male	11005	14856	20061	26397
African	Female & Male	6080	8184	10952	14259
Coloured	Female & Male	1040	1398	1880	2457
Indian	Female & Male	640	862	1162	1523
White	Female & Male	3245	4411	6068	8159
Overall	Female	7576	10303	14053	18644
African	Female	4052	5492	7447	9830
Coloured	Female	714	966	1313	1725
Indian	Female	450	611	828	1090
White	Female	2360	3233	4466	5998
Overall	Male	3429	4553	6008	7754
African	Male	2028	2692	3505	4429
Coloured	Male	326	432	567	732
Indian	Male	190	252	335	433
White	Male	885	1177	1602	2160
SENSITIVITY ANALYSIS using (corrected) World Bank estimates					
Overall	Female & Male	11065	14927	20181	26570
African	Female & Male	6105	8213	11000	14327
Coloured	Female & Male	1043	1402	1886	2467
Indian	Female & Male	644	867	1170	1535
White	Female & Male	3273	4445	6124	8241
Overall	Female	7623	10358	14148	18778
African	Female	4073	5516	7488	9887
Coloured	Female	717	970	1318	1734
Indian	Female	453	615	834	1100
White	Female	2380	3258	4507	6057
Overall	Male	3442	4569	6033	7792
African	Male	2032	2697	3512	4440
Coloured	Male	326	433	568	734
Indian	Male	191	252	336	435
White	Male	893	1187	1617	2184

* rounded to nearest whole number

Supplementary Figure 1: South Africa census population vs. United Nations estimated population prospects for year 2011

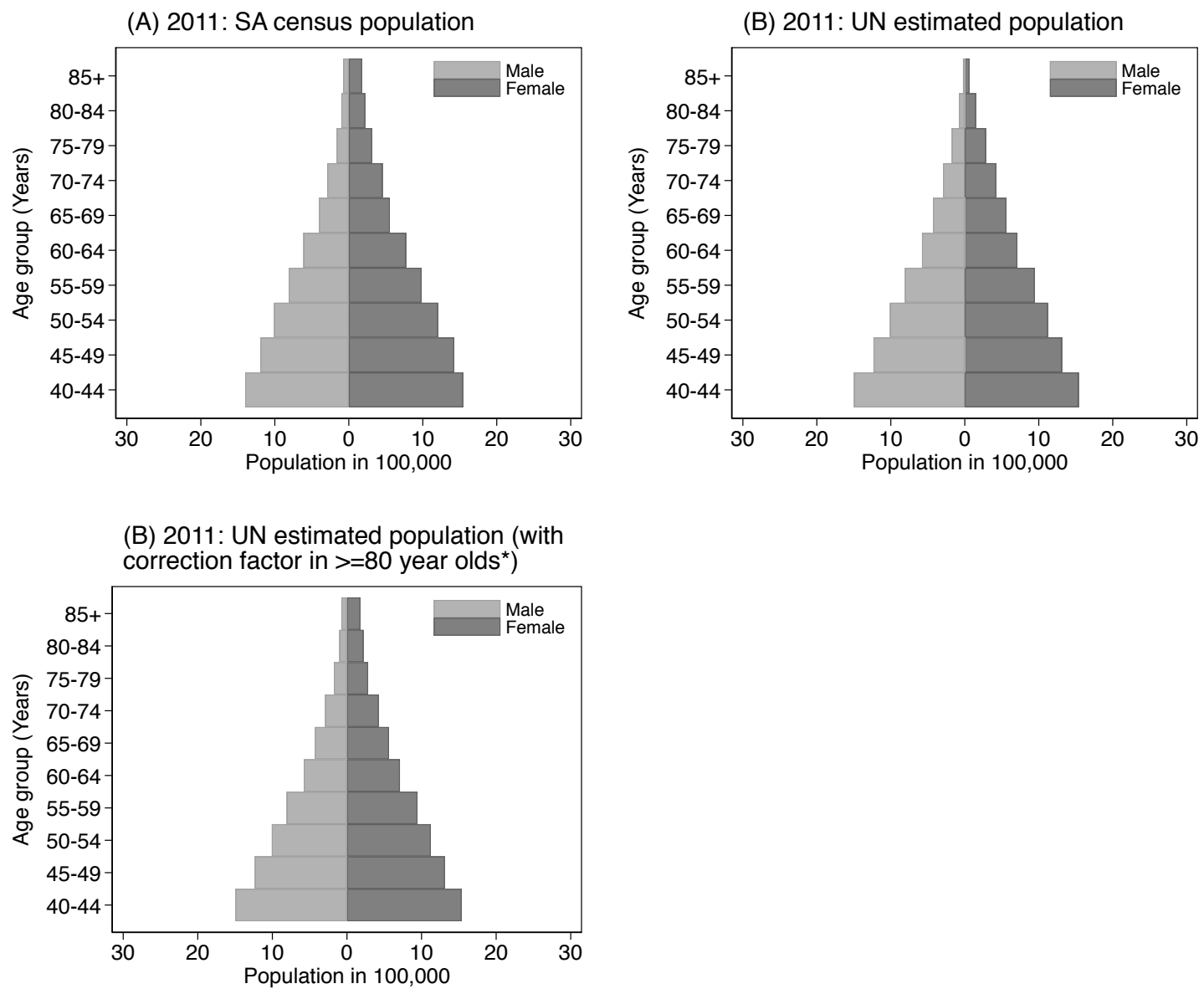

*Correction factor: 1.47 (females 80-84); 2.81 (females 85+); 1.27 (males 80-84); 3.04 (males 85+)

Supplementary Figure 2: South Africa census population vs. World Bank estimated population prospects for year 2011

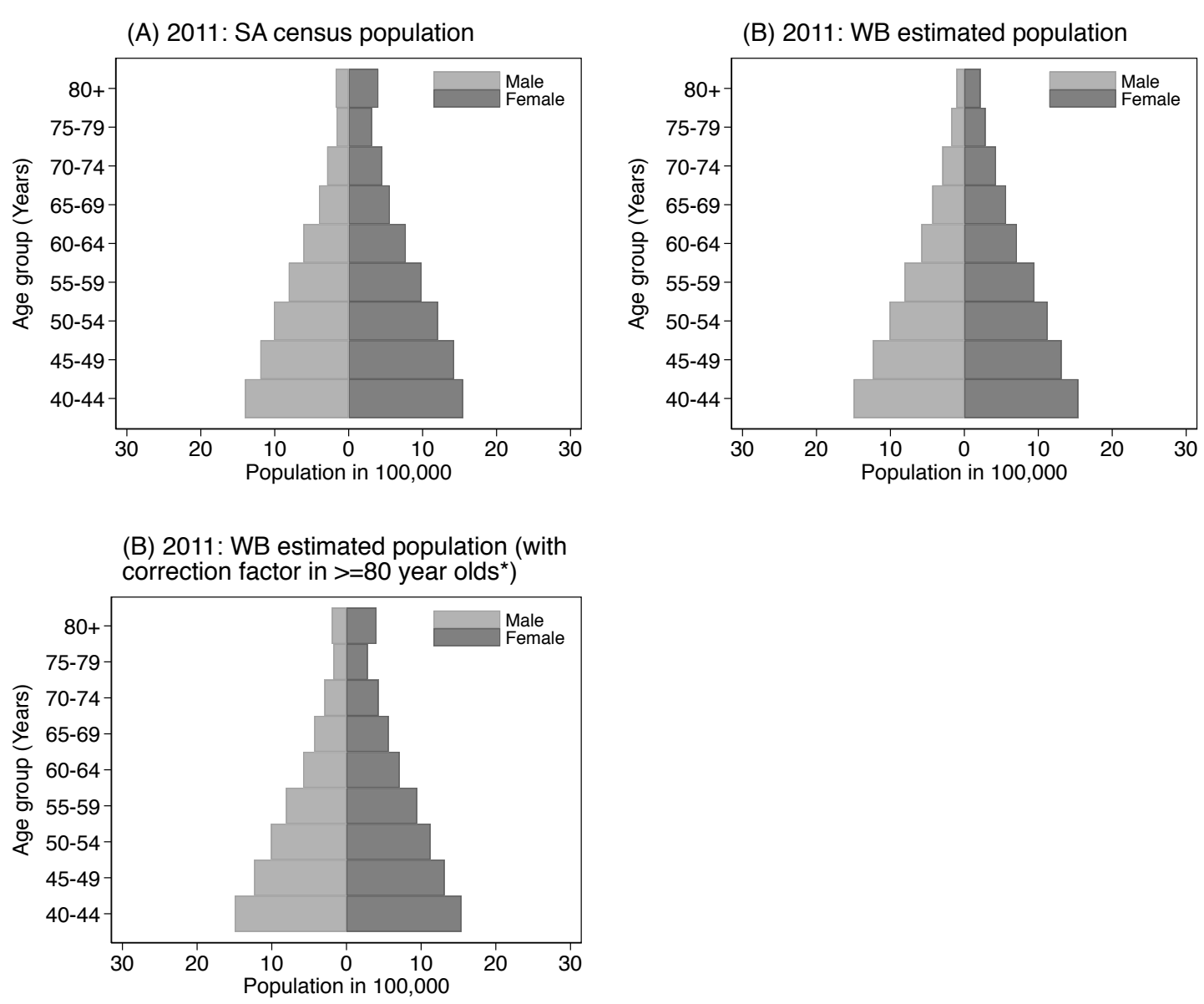

*Correction factor: 1.86 (females 80+); 1.68 (males 80+)
